# Estimating the Prevalence of Schizophrenia in the General Population of Japan Using an Artificial Neural Network–Based Schizophrenia Classifier: Web-Based Cross-Sectional Survey

**DOI:** 10.2196/66330

**Published:** 2025-01-29

**Authors:** Pichsinee Choomung, Yupeng He, Masaaki Matsunaga, Kenji Sakuma, Taro Kishi, Yuanying Li, Shinichi Tanihara, Nakao Iwata, Atsuhiko Ota

**Affiliations:** 1 Department of Public Health, Fujita Health University School of Medicine, 1-98 Dengakugakubo, Kutsukake-cho, Toyoake, 470-1192, Japan, 81 562-93-2476, 81 562-93-3079; 2 Department of Psychiatry, Fujita Health University School of Medicine, Toyoake, Japan; 3 Department of Public Health and Health Systems, Nagoya University Graduate School of Medicine, Nagoya, Japan; 4 Department of Public Health, Kurume University School of Medicine, Kurume, Japan

**Keywords:** schizophrenia, schizophrenic, prevalence, artificial neural network, neural network, neural networks, ANN, deep learning, machine learning, SZ classifier, web-based survey, epidemiology, epidemiological, Japan, classifiers, mental illness, mental disorder, mental health

## Abstract

**Background:**

Estimating the prevalence of schizophrenia in the general population remains a challenge worldwide, as well as in Japan. Few studies have estimated schizophrenia prevalence in the Japanese population and have often relied on reports from hospitals and self-reported physician diagnoses or typical schizophrenia symptoms. These approaches are likely to underestimate the true prevalence owing to stigma, poor insight, or lack of access to health care among respondents. To address these issues, we previously developed an artificial neural network (ANN)–based schizophrenia classification model (SZ classifier) using data from a large-scale Japanese web-based survey to enhance the comprehensiveness of schizophrenia case identification in the general population. In addition, we also plan to introduce a population-based survey to collect general information and sample participants matching the population’s demographic structure, thereby achieving a precise estimate of the prevalence of schizophrenia in Japan.

**Objective:**

This study aimed to estimate the prevalence of schizophrenia by applying the SZ classifier to random samples from the Japanese population.

**Methods:**

We randomly selected a sample of 750 participants where the age, sex, and regional distributions were similar to Japan’s demographic structure from a large-scale Japanese web-based survey. Demographic data, health-related backgrounds, physical comorbidities, psychiatric comorbidities, and social comorbidities were collected and applied to the SZ classifier, as this information was also used for developing the SZ classifier. The crude prevalence of schizophrenia was calculated through the proportion of positive cases detected by the SZ classifier. The crude estimate was further refined by excluding false-positive cases and including false-negative cases to determine the actual prevalence of schizophrenia.

**Results:**

Out of 750 participants, 62 were classified as schizophrenia cases by the SZ classifier, resulting in a crude prevalence of schizophrenia in the general population of Japan of 8.3% (95% CI 6.6%-10.1%). Among these 62 cases, 53 were presumed to be false positives, and 3 were presumed to be false negatives. After adjustment, the actual prevalence of schizophrenia in the general population was estimated to be 1.6% (95% CI 0.7%-2.5%).

**Conclusions:**

This estimated prevalence was slightly higher than that reported in previous studies, possibly due to a more comprehensive disease classification methodology or, conversely, model limitations. This study demonstrates the capability of an ANN-based model to improve the estimation of schizophrenia prevalence in the general population, offering a novel approach to public health analysis.

## Introduction

Schizophrenia is a complex psychiatric disorder characterized by significant impairments in the perception of reality and behavioral changes. It includes positive (eg, delusions, hallucinations, disorganized thinking, and notably disorganized behaviors) and negative symptoms (eg, limited speech, decreased emotional expression, inability to experience interest or pleasure, and social withdrawal). Affected individuals often experience cognitive impairments affecting memory, attention, and problem-solving abilities [1,2]. The Global Burden of Disease Study [3] reported that schizophrenia accounted for 23.6 million estimated cases in 2019, with an age-standardized prevalence rate of 287.4 per 100,000 people. Median estimates from internationally published studies indicate a 12-month prevalence of 0.33%, lifetime prevalence of 0.48%, and point prevalence of 0.32% [4]. However, there is a lack of published studies on the prevalence of schizophrenia in the general Japanese population. Only a few population-based studies have estimated the prevalence of schizophrenia in Japan. For instance, the point prevalence of schizophrenia stratified by sex and age ranges from 0.4% to 1.1% [5]. However, these figures were derived from the Vital Statistics and the Patient Survey in 2008, which probably do not represent the general population since the Patient Survey could not encompass patients who do not visit a doctor. The estimated lifetime prevalence of schizophrenia is 0.59%, according to a secondary analysis of the 2019 cross-sectional Japan National Health and Wellness Survey [6]. However, this study may produce underestimation since it only depended on physician diagnoses. Individuals with schizophrenia hardly self-identify as having schizophrenia because of stigma and lack of awareness of schizophrenia and its symptoms [7-13].

In our previous research, we developed an artificial neural network (ANN)–based schizophrenia classification model (SZ classifier) to classify schizophrenia cases in the Japanese population and verified its generalizability [14]. The model was trained by using data from a large-scale Japanese web-based survey; the presence of schizophrenia served as a response variable. The general information including demographics, health-related background, physical comorbidities, psychiatric comorbidities, and social comorbidities were collected and used as feature variables. This SZ classifier achieved high internal validity with a high area under the receiver operating characteristic curve (AUC) of 0.86 [14]. In another study, we also assessed the SZ classifier’s external validity by applying it to psychiatric outpatients, achieving a sensitivity of 75% [15]. The SZ classifier suggests a potential for classifying schizophrenia cases in the general Japanese population.

In this study, we aimed to estimate the prevalence of schizophrenia in the general Japanese population using the SZ classifier through the following three approaches: (1) conducting a population-based survey to recruit participants and collect their general information, (2) sampling participants who match the distribution to the population demographic structure, and (3) using a pretrained machine learning model to detect schizophrenia cases. Study participants were randomly sampled from a pooled panel. The samples are matched to the demographic distribution of the Japanese population. General information, the same as that for the development of the SZ classifier, was collected through the web-based survey for each study participant. The estimation of the schizophrenia prevalence was implemented through the following two steps: (1) calculating the crude prevalence based on crude cases that are detected by the SZ classifier and (2) refining the crude prevalence by excluding false-positive estimates and including the false-negative estimates.

## Methods

### Development of the SZ Classifier

The SZ classifier was developed as part of a prior study [14] using a dataset obtained from an internet research agency’s pooled panels (Rakuten Insight, Inc) [16]. The dataset included 223 individuals with schizophrenia and 1776 healthy controls. For each sample, schizophrenia status served as the response variable, while demographic details, health-related background information, and physical, psychiatric, and social comorbidities were considered feature variables. Evidence on the association between these feature variables and schizophrenia has been previously published [17,18]. For example, demographic and health-related characteristics included age, BMI, smoking status, restriction in functional capacity, and bowel frequency. Physical comorbidities included overweight, cancer, cardiovascular disease, heart failure, and hypertension. Psychiatric comorbidities included frequency of sleep medication use, bedtime, hours of sleep, perceived stress, and positive reasons for living. The social comorbidities included educational background, type of occupation, type of employment, household income, and marital status. The inclusion criteria of the study participants and definitions of the feature variables are detailed in previous publications [14,18] and provided in Multimedia Appendix 1.

The SZ classifier was constructed using an ANN concept. This multilayer perceptron neural network model comprised 5 hidden layers with 128, 64, 32, 16, and 8 neurons in each respective layer. It used the HeNormal weight initializer and the ReLU activation function in the hidden layers. A sigmoid activation function was used in the output layer. Comprehensive details can be found in a prior publication [14]. The model’s performance was evaluated using the AUC score. During internal validation, the SZ classifier achieved an AUC of 0.86, accuracy of 0.93, specificity of 0.96, and sensitivity of 0.56. He et al [14] performed 10,000 bootstrapping iterations and a 10-fold cross-validation to further evaluate the algorithm’s performance and achieved AUCs of 0.847 and 0.81, respectively.

### External Validation of the SZ Classifier

External validation of the SZ classifier was conducted by applying it to a sample of patients who visited the psychiatric department of Fujita Health University Hospital between January 2022 and May 2023 [15]. Patients aged 20‐75 years diagnosed with schizophrenia, major depressive disorder (MDD), bipolar disorder (BD), or obsessive-compulsive disorder (OCD) were selected. Therefore, 150 participants were included in the study: 61 with schizophrenia, 56 with MDD, 32 with BD, and one with OCD. The participants were required to complete the same survey used to develop the SZ classifier to predict the likelihood of schizophrenia. The external validation process revealed that the SZ classifier achieved a sensitivity of 0.75 but misclassified 55.4% of patients with MDD and 59.4% of patients with BD as having schizophrenia [15].

### Estimation of the Prevalence of Schizophrenia by Using the SZ Classifier

A cross-sectional study was conducted using data from a large-scale web-based survey in Japan. The number of participants was set to 750. The sample size was decided using the single population proportion formula (n=[Z^2^∙p(1–p)]/e^2^) by assuming a 95% CI (Z=1.96), 1% margin of error (e=0.01), and 2% proportion of schizophrenia (p=2%) based on previous studies. Participants were randomly recruited from those registered as survey monitors with Rakuten Insight, a large-scale web-based survey incorporated approximately 2.2 million registered members as of September 2022 and provided reliable data with regular quality maintenance [16]. Specifically, the recruited criteria were set to the combination of 3 items of age intervals (20‐75 years old, divided into 5 distinct age groups), gender (male and female), and regions (13 geographical administrative regions in Japan: Hokkaido, Tohoku, North Kanto, Greater Tokyo Area, Koshinetsu, Hokuriku, Tokai, Kinki excluding Keihanshin, Keihanshin, Chugoku, Shikoku, Kyushu excluding Okinawa, and Okinawa). For each combination category of these 3 items, the number of participants was set to match the composition of the national population as of October 1, 2021, as estimated by the Ministry of Internal Affairs and Communications, Japan. Participants were recruited until the target number for each combination category was reached. The recruitment period was from November 20 to 26, 2023. This was a web-based survey in which only authorized panelists were allowed to respond to the survey to prevent fraudulent registrants from participating. To administer the survey, respondents had to log in to their accounts to prevent multiple entries from the same individual. They were required to answer 38 questions (some questions had several subquestions) that are distributed into 38 pages. Respondents were asked to review their responses and ensure completeness before submission. Specifically, the web-based survey system notified respondents if there were any blank answers. The participation rate for this survey was estimated to be 0.30, and the completion rate was not calculated because if the respondents terminated during the answering process (ie, logged out, closed the web page, etc), they were not counted in the statistics. Incomplete questionnaires or questionnaires that were submitted too soon were not used.

Each participant’s demographic information, health-related backgrounds, and physical, psychiatric, and social comorbidities were collected and applied to the SZ classifier, yielding the predicted crude number of schizophrenia cases and crude prevalence of schizophrenia. Textbox 1 shows a summary of the feature variable categories collected from study participants. These variables are the same as those used in developing the SZ classifier. For detailed information on these variables, please refer to Multimedia Appendix 1.

Textbox 1. **Summary of categories of feature variables collected from study participants, including demographic, health-related backgrounds, physical comorbidities, psychiatric comorbidities, and social comorbidities.**

**Demographic, health-related backgrounds, and physical comorbidities:**

- Age
- BMI
- Smoking
- Alcohol drinking
- Sports
- Eating behaviors
- Bowel movement
- Restriction in functional capacity
- Self-rated health status
- Physical comorbidities

**Psychiatric comorbidities:**

- Frequency of sleep medication use
- Bedtime
- Sleep hours and qualities
- Depressive symptoms
- Perceived stress
- Positive reason for living (ikigai)
- Internet use hours per week

**Social comorbidities:**

- Regularity of health checkups
- Educational background
- Household income
- Type of employment and occupation
- Marital status and family structure
- Social support and social capital

We further refined these crude estimates by excluding false positives and adding false negatives. This was based on the model’s internal and external validation results and literature reviews. Details of the model’s internal and external validation results are provided in the Multimedia Appendix 1.

False-positive cases included individuals without mental disorders who were misclassified as having schizophrenia and those with other mental disorders who were misclassified as having schizophrenia. The false-positive cases were calculated as follows:


$$N_{fp}=\left(P_{hc}\cdot r_{hc}+P_{MDD}\cdot r_{MDD}+P_{BD}\cdot r_{BD}\right)\cdot n$$

where N_fp_ represents the number of false-positive cases, P_hc_ represents the percentage of individuals who have not had any mental disorders in the population, r_hc_ represents the rate at which the SZ classifier falsely classified individuals without mental disorders as having schizophrenia, P_MDD_ represents the prevalence of MDD in the population, r_MDD_ represents the rate at which the SZ classifier falsely classified individuals with MDD as having schizophrenia, P_BD_ represents the prevalence of BD in the population, r_BD_ represents the rate at which the SZ classifier falsely classified individuals with BD as having schizophrenia, n represents the sample size, which included 750 participants.

The SZ classifier falsely classified 3.1% of the individuals without mental disorders as having schizophrenia [14]. The SZ classifier misclassified 55.4% of the patients with MDD and 59.4% of the patients with BD as having schizophrenia [15]. According to a literature review, 80% of the general population in Japan does not have mental disorders [19]. The point prevalence of MDD and BD was 7.9% [20] and 0.4% [21], respectively; namely, P_hc_=80%, r_hc_=3.1%, P_MDD_=7.9%, r_MDD_=55.4%, P_BD_=0.4%, r_BD_=59.4%, and n=750.

False-negative cases included individuals with schizophrenia but were not classified as schizophrenia cases. The proportion of true-positive to false-negative cases in the external validation was 46:15 [15]. Hence, the false-negative cases can be calculated as follows:


$$N_{tp}=N_{crude}-N_{fp}$$

$$N_{tp}/N_{fn}=p$$

where N_tp_ represents the cases predicted by the SZ classifier, N_crude_ represents the crude number of schizophrenia cases estimated by the SZ classifier, N_fn_ represents the false-negative cases, and p represents the proportion of true-positive cases to false-negative cases in the external validation, which was 46:15 [15].

**Figure 1.**
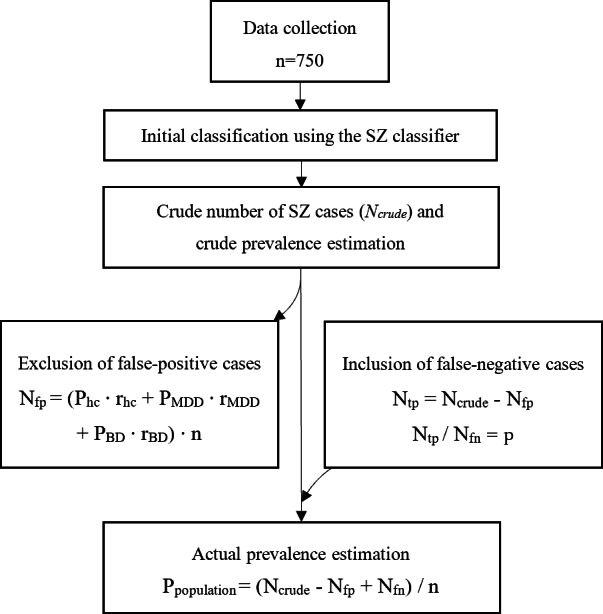
Flowchart illustrating the data analysis and refinement process for estimating the prevalence of schizophrenia in the general Japanese population. Data were collected from 750 participants during November 20 to 26, 2023. Participants’ demographic information, health-related background, and physical, psychiatric, and social comorbidities were assessed using the schizophrenia classification model to initially estimate the crude number of schizophrenia cases and crude prevalence. The refinement process involved excluding false-positive cases and including false-negative cases, resulting in an actual estimation of schizophrenia prevalence in the general population of Japan.

Finally, the prevalence of schizophrenia in the population could be calculated by


$$P_{population}=\left(N_{crude}-N_{fp}+N_{fn}\right)/n$$

where P_population_ represents the prevalence of schizophrenia in the population (Figure 1).

### Statistical Analysis

The 95% CIs were calculated from 10,000 bootstrap replicates. Statistical analyses were performed using Python (version 3.8; Python Software Foundation), with a Jupyter Notebook (Project Jupyter) as a coding notebook.

### Ethical Considerations

The study protocol was reviewed and approved by the institutional review board at Fujita Health University (HM23-253). All procedures were performed following the ethical standards of the Declaration of Helsinki. The internet survey agency respected the Act on the Protection of Personal Information in Japan. Informed consent was obtained at the initial web page of the survey, where the purpose of the survey and the instructions to the respondents were displayed. Only those who had read the survey’s purpose and instructions and provided informed consent by clicking the agreement button could proceed with the survey. Participants for the external validation of the SZ classifier were diagnosed by experienced psychiatrists. The data were anonymous and deidentified before being provided to researchers. This was a voluntary survey. Participants received Rakuten Points, which can be used for Rakuten services such as web-based or offline shopping. No image data were collected so that there were no concerns about identifying individual participants.

## Results

Of the 750 samples analyzed, 62 were classified as schizophrenia cases (N_crude_). The crude prevalence of schizophrenia in the general population of Japan, as estimated using the SZ classifier, was 8.3% (95% CI 6.6%-10.1%; Table 1). Among these 62 cases, 53 were presumed to be false positives, and 3 were presumed to be false negatives. After adjustments, the actual prevalence of schizophrenia in the general population was estimated to be 1.6% (95% CI 0.7%-2.5%; Table 2).

**Table 1.** Results of crude estimates of schizophrenia cases by the schizophrenia classification model (SZ classifier), crude schizophrenia prevalence, false-positive estimates, and true-positive estimates.

	Cases, n	Prevalence, %
**Crude estimates of predicted schizophrenia by the SZ classifier**	62	8.3 (6.6-10.1)^a^
False positive: no mental disorders misclassified as schizophrenia	18	2.5
False positive: patients with MDD^b^ misclassified as schizophrenia	33	4.4
False positive: patients with BD^c^ misclassified as schizophrenia	2	0.2
True positive: patients with schizophrenia who were accurately classified as schizophrenia cases	9	1.2

a 95% CI value.

b MDD: major depressive disorder.

c BD: bipolar disorder.

**Table 2.** Confusion matrix and adjusted prevalence of schizophrenia in the general population of Japan.

		Adjusted cases estimation	
		Schizophrenia	NonSchizophrenia
**Predicted cases, n**			
	Schizophrenia	9	53
	Nonschizophrenia	3	685
	Total	12	738
Prevalence, % (95%CI)		1.6 (0.7‐2.5)	―^a^

a Not applicable.

## Discussion

### Principal Results

Our findings indicate that the estimated prevalence of schizophrenia in the general population of Japan using the SZ classifier was 1.6% (95% CI 0.7%-2.5%). This estimate is slightly higher than those in previously published figures [5,6]. This discrepancy could be attributed to several reasons, including the possibility that our SZ classifier estimates lifetime prevalence rather than point prevalence, a potentially more comprehensive disease classification methodology, and calculating the actual prevalence by referencing various prevalence data from diverse published sources.

To ensure the validity of the data used in our study, 3 main points were considered. The first point is the trustworthiness of the data source. The data were collected and managed by Rakuten Insight Panel, a large-scale web-based survey panel. The panel’s quality has been maintained by company procedures and has been widely used by research institutions in Japan for population-based studies [16]. The second point is the appropriateness of the data used for model development. All feature variables were decided both through a literature review [17] and studies demonstrating that all these variables effectively differentiate between individuals with and those without schizophrenia [14,15,18]. Additionally, all the schizophrenia cases were defined by using a screening protocol instead of relying solely on the self-reported illness conditions. Schizophrenia cases had to meet the following four criteria: (1) currently having schizophrenia only, schizophrenia and migraine, schizophrenia and a sleep disorder, or schizophrenia, migraine, and a sleep disorder; (2) having experienced auditory hallucinations lasting more than 1 month; (3) not having used stimulants or other illegal drugs and not having been an alcoholic; and (4) having experienced the first auditory hallucination lasting more than 1 month at less than 60 years of age [18]. Third, the high performance of the developed model relies on high-quality data. The robustness and generalizability of the model were confirmed through good internal validation (AUC=0.86) [14] and external validation results (sensitivity=0.75) [15].

The SZ classifier predicts schizophrenia status using various feature variables. Each feature has been reported as being significantly associated with schizophrenia in previous studies [14,15,17,18]. By training the model with these features and applying data from the study participants, the probability of schizophrenia can be calculated. Although this probability may not be highly interpretable for diagnosis on an individual level, it reliably represents the proportion of specific cases within a target group. When the model demonstrates good generalizability, this proportion can be extended to estimate prevalence by considering the false positives and false negatives on a population level.

According to the feature importance ranking estimated by the model, social comorbidities, such as household income, type of employment, educational background, and type of occupation, play significant roles [14]. These factors may have been influenced by previous psychiatric conditions, which could have had long-term effects on the data used in this analysis. For instance, individuals diagnosed with schizophrenia may have had to stop attending school or leave their jobs, affecting their current income and occupation [22]. Consequently, the model might have predicted them as having schizophrenia even though they had recovered, which is consistent with the lifetime prevalence estimates. Several studies have shown that the prevalence of schizophrenia increases when it extends from one point to the lifetime of an individual [4,23].

To the best of our knowledge, this is the first prevalence study to apply an ANN model as a schizophrenia case classifier using web-based survey data from the general population of Japan. ANNs, which are computational models inspired by the inner processes of the human brain, are increasingly used in public health research [24,25]. Technically, ANNs are excellent at capturing complex and nonlinear relationships within large datasets. Hence, models constructed using ANNs perform better than classical methods based on linear equations [26,27]. Several studies have proposed ANN models or machine learning techniques to detect and classify schizophrenia [28,29]. These studies developed models using large amounts of clinical data (ie, magnetic resonance images, positron emission tomography scans, electroencephalography, and subjective patient posture interpretation). These studies have applied machine learning techniques to improve detection and diagnosis and aid in clinical decision-making. Bae et al [30] demonstrated the use of machine learning to detect signs of schizophrenia by analyzing social media texts, supporting the role of machine learning models in improving symptoms monitoring and potentially early detection of schizophrenia.

The use of ANN models to estimate prevalence has been reported in previous studies. For example, suicidal ideation risk prediction models were developed based on demographic, behavioral, and psychological data obtained from computer-based questionnaires. These models were then used to estimate the prevalence of suicidal ideation among Chinese college students. The models demonstrated a prediction accuracy of over 91.7%, and the AUC score increased to 0.96 [31]. Compared with previous studies, our SZ classifier has undergone external validation to demonstrate its generalizability [15]. This study supports the use of machine learning–based methods for prevalence estimation.

This study design may provide evidence to overcome biases encountered in traditional self-identifying surveys due to stigma, poor insight, and lack of access to health professionals experienced in conventional self-identifying surveys [32,33]. Stocking et al [34] reported that they first used a mathematical model to explore the correlation between several comorbidities and a specific illness in a small sample, and then applied this correlation to a larger population to identify previously underreported cases. They noted that this approach is effective for addressing the underreporting of various chronic conditions associated with social stigma, challenges in detection and treatment, or inconsistent treatment engagement (eg, mental disorders, obesity, hypertension, sleep disorders, etc). In this study, schizophrenia cases were estimated by incorporating general information, including demographics, health-related backgrounds, and physical, psychological, and social comorbidities, to calculate the likelihood of schizophrenia. This approach encompasses a broader range of patients in the general population than previous methodologies that relied on clinical records or typical schizophrenia symptoms. In contrast to a diagnosis that depends on the self-reporting of typical schizophrenia symptoms, which requires participants to openly acknowledge symptoms that may suggest schizophrenia, our methodology provides a more objective assessment. Patients with schizophrenia are often stigmatized and not widely accepted in society, leading to embarrassment and hesitation in disclosing their symptoms and avoiding negative perceptions. Stigmatizing attitudes related to mental illness have been reported to prevent individuals from seeking counseling [35] and harm the self-esteem of people with serious mental illnesses [36]. Additionally, the wording of questionnaires that focus on negative aspects may cause respondents to feel negative and skip or conceal their actual symptoms, resulting in underreported results in self-identifying surveys [37,38]. By using general information, our SZ classifier mitigated these biases, potentially providing a more comprehensive estimate of patients with schizophrenia in Japan.

The SZ classifier has the advantage of potentially detecting patients with schizophrenia who do not perceive subjective symptoms. Hoffman et al [39] reported that a common reason why people with schizophrenia lack insight into their illness is that their ability to objectively assess their own pathological experiences is impaired. By using objective indicators such as demographics, health-related backgrounds, and physical, psychological, and social comorbidities, the SZ classifier showed a potential ability of detecting patients who do not perceive subjective symptoms and estimating the prevalence of schizophrenia in the population. This study may offer unique advantages over previous estimation methods based solely on medical data.

The higher prevalence estimated in our study suggests that schizophrenia might be more common than previously believed in the Japanese population. This finding confirmed our hypothesis regarding the underestimation of schizophrenia cases in the Japanese population. These findings have significant implications for public health management and resource allocation. Greater knowledge about the actual prevalence of schizophrenia can result in enhanced mental health services and increased support systems for managing this public health concern.

### Limitations

Our study has several limitations. First, the actual prevalence of schizophrenia was calculated by referencing various prevalence data from diverse published sources, including the prevalence of individuals who did not have any mental disorder, the prevalence of those who had MDD, and the prevalence of those who had BD. Using data from different studies, which may have used different methodologies and population samples, causes uncertainty in the data, thereby affecting the estimated values. Second, although the data used in this study were collected from an extensive web-based survey that efficiently integrates large amounts of data, it may introduce a selection bias toward those with internet access. Those who are less likely to participate in web-based surveys, such as individuals without internet access, institutionalized patients, patients with severe comorbidities, older adults, and poor people, might be underrepresented. Third, the SZ classifier, designed to capture a broad range of patients, may include individuals with subclinical symptoms or comorbid conditions similar to schizophrenia. Despite efforts to rule out incorrectly classified cases, there is a chance that other mental disorders will not be sufficiently considered. This inclusion may lead to an overestimation of the true prevalence of schizophrenia. Fourth, for mental disorders comorbid with schizophrenia symptoms (eg, schizoaffective disorder, characterized by symptoms of both schizophrenia and mood disorders, etc), these cases were not included in the SZ classifier validation. Also, due to the complexity of mental disorders, the diagnosis of a disease without confirmation from a physician may seem arbitrary. Therefore, these results should be interpreted with caution.

### Conclusions

This study applied a pretrained SZ classifier to estimate the prevalence of schizophrenia in a Japanese population. The estimated results are slightly higher than those of previous studies. Considering the shortcomings of focusing on a single type of mental disorder and the need to increase the sample size, this study shows promise for prevalence-estimation research. Potential future research directions may focus on developing models to classify other mental disorders, such as BD and MDD, as well as estimate prevalence. These findings are expected to support the versatility of pretrained models in epidemiological research.

## Supplementary material

10.2196/66330 Multimedia Appendix 1 Inclusion criteria, variable definition, and model validation results.

## Abbreviations

ANN: artificial neural network
AUC: area under the receiver operating characteristic curve
BD: bipolar disorder
MDD: major depressive disorder
OCD: obsessive-compulsive disorder
SZ classifier: schizophrenia classification model
