# Supplementary material for: Estimating the Prevalence of Schizophrenia in the General Population of Japan Using an Artificial Neural Network–Based Schizophrenia Classifier: Web-Based Cross-Sectional Survey
Source: JMIR Form Res. 2025 Jan 29;9:e66330. doi: 10.2196/66330 (PMC11798565; doi:10.2196/66330)
Supplement: Multimedia Appendix 1 [file formative-v9-e66330-s001.docx]

Multimedia Appendix 1

1. **Schizophrenia classification (SZ classifier) model development**

**Inclusion criteria of study participants**

Data were collected from the pooled panel of an internet research agency (Rakuten Insight, Inc., Tokyo, Japan) [1]. Individuals who participated in the study answered a web-based survey. Participants’ age was limited from 20 to 75 years old.

For participants who currently have schizophrenia, we sampled 5,584 people who enduring having schizophrenia in the Rakuten Insight disease panel, 3,256 respondents answered the following four questions before the survey: (1) Are you currently enduring with schizophrenia only, or schizophrenia and migraine, or schizophrenia and sleep disorder, or schizophrenia, migraine, and sleep disorder? (2) Have you experienced auditory hallucinations that persisted for more than a month? (3) Have you completely abstained from using stimulants or other illegal drugs and never had an alcohol addiction? (4) Have you ever had your first auditory hallucination lasting more than a month before the age of 60? Respondents who answered "Yes" to all four questions were classified as currently having schizophrenia. Consequently, 223 individuals participated in the survey.

For participants who do not currently have schizophrenia, we sampled 28,000 people in the Japan COVID-19 and Society Internet Survey (JACSIS) and the Japan Society and New Tobacco Internet Survey (JASTIS) which also conducted by the Rakuten Insight disease panel [2-4], 6,656 respondents answered the following four questions before the survey: (1) Are you currently experiencing any mental illness? (2) Have you had a mental illness in the past? (3) Have you ever experienced auditory hallucinations? (4) Have you ever used stimulants or other illegal drugs, struggled with alcoholism, or received psychiatric treatment? Respondents who answered "No" to all four questions were classified as not having schizophrenia. Consequently, 1,776 individuals participated in the survey.

In summary, 223 participants with schizophrenia and 1,776 healthy controls, aged 20 to 75 years, were included in the study.

**Definitions of feature variables**

Individual participants answered the self-administered questionnaire estimated 1) demographic detail, health-related backgrounds, and physical comorbidities, 2) psychiatric comorbidities, and 3) social comorbidities.

**1) Demographic, health-related backgrounds, and physical comorbidities**

Demographic, health-related backgrounds, and physical comorbidities include **age, body mass index** (BMI), **smoking status** [current, past, never], **number of cigarettes per day**, **alcohol drinking frequency** [never, past, current: <1 day in one month; 1-3 days in one month; 1-2 days in one week; 3-4 days in one week; 5-6 days in one week; everyday.], **sports** [frequency: < once in one month; 1-3 times in one month; 1-2 times in one week; 3-4 times in one week; almost every day. Intensity: each time < 30 minutes; 30-59 minutes; 1-2 hours; 2-3 hours; 3-4 hours; >4 hours], **eating behaviors** [tendency to overeat: yes/no. Eating speed: very fast; fast; normal; slow; very slow. Having breakfast, eating out, and eating instant foods: < once in one month; 1-3 times in one month; 1-2 times in one week; 3-4 times in one week; almost every day], **bowel movement** [frequency: <3times per week; 3-4 times per week; 5-6 times per week; once a day; > once a day], **Stool** [soft; normal; hard; recurrent diarrhea and constipation], **restriction in functional capacity** [5], **self-rated health status** (SRHS) [6], and **physical comorbidities** [overweight, cancer, cardiovascular disease, heart failure, hypertension, diabetes, dyslipidemia, gout, sleep apnea syndrome, and fracture: have been treated or not].

**Restriction in functional capacity**

To determine functional capacity restrictions, we used the Scale of Independence in Daily Living for the Disabled Elderly published by the Ministry of Health, Labour and Welfare, Japan [5]. The term ”restrictions in functional capacity” refers to a multidimensional concept that involves sensory loss, impaired mobility, vascular disorders, gait impairments, problems with daily living (ADLs), and changes in body systems. Participants self-assessed restrictions by choosing one of the following options: (1) “I have no physical disabilities,” (2) “I go out alone, using transportation,” (3) “I can only go out alone in my neighborhood,” (4) “I go out with help and live mostly out of bed during the day,” (5) “I can go out with help, but I go out infrequently, and I spend most of the daytime sleeping on and off in bed,” (6) “I can ride in a wheelchair by myself and eat and toilet away from the bed,” (7) “I can ride in a wheelchair with assistance.,” (8) “I can roll over in bed,” and (9) “I cannot roll over in bed.”

**Self-rated health status (SRHS)**

SRHS is a self-reported measure of health status that incorporates a person’s biological, mental, social, and functional aspects, including individual and cultural beliefs and health behaviors. It is a strong predictor of all-cause mortality in general populations [6]. Participants responded to the question “What do you think of your general health status during the previous month?” by choosing one of the following options: “great,” “pretty good,” “good,” “not so good,” and “bad.”

**2) Psychiatric comorbidities**

Psychiatric comorbidities include **frequency of sleep medication use** [never; < 1 day per week; 1-2 days per week; 3-4 days per week; 5-6 days per week; every day], **bedtime** [before 7:00pm; around 8:00pm; around 9:00pm; around 10:00pm; around 11:00pm; around 0:00am; around 1:00am; around 2:00am; around 3:00am; after 4:00am; not fixed], **sleep hours** [≤5 hours; 6 hours; 7 hours; 8 hours; 9 hours; ≥10 hours], **hypnagogic disorder frequency** [almost never; < 1 time per week; 1-2 times per week; 3-4 times per week; 5-6 times per week; almost every day], **deep sleep disorder frequency** (waking up tired) [almost never; < 1 time per week; 1-2 times per week; 3-4 times per week; 5-6 times per week; almost every day], **middle wakening, or early wakening frequency** [almost never; < 1 time per week; 1-2 times per week; 3-4 times per week; 5-6 times per week; almost every day], **depressive symptoms** [7,8], **perceived stress** [9], ***ikigai*** (a Japanese term which means positive reason for living), **happiness**, and **internet use hours per week**.

**Depressive symptoms (CES-D)**

We used a modified 11-item Center for Epidemiological Studies Depression (CES-D) Scale in this study [7,8]. The existence of depressive symptoms was defined as a score of 8 or higher.

**Perceived stress (PSS-4)**

We assessed perceived stress with a 4-item Perceived Stress Scale (PSS-4) [9]. Scores are on a 16-point scale, with higher total scores indicating more severe perceived stress. Perceived stress was defined as being present when the score was higher than 7, the median of the PSS-4 scores for non-schizophrenic participants.

**Ikigai**

The Japanese term “Ikigai” is a positive reason for living. Participants were asked, “Do you have any positive reasons to live?” and answered the question from four options: “very much so,” “yes,” “no,” or “not at all.” Participants who answered “no” or “not at all” were defined as absent of ikigai.

**Happiness**

Participants were asked, “How happy do you feel about your life?” and answered the question from four options: “very happy,” “happy,” “neither happy nor unhappy,” and “unhappy.” Participants who answered “neither happy nor unhappy” or “unhappy” were defined as absence of happiness.

**3) Social comorbidities**

Social comorbidities include **taking regular health checkups** [yes/no], **educational background** [junior/senior high school; university; junior college; or vocational school], **type of occupation** [unemployed; homemaker; white-collar workers; or blue-collar workers], **type of employment** [regular; irregular; or self-employed/business people], **household income** [< 3; 3–6; 6–9; 9-12; 12-15; ≥ 15 million Japanese Yen], **marital status** [unmarried; married; divorced; widowed; others], **family structure** [living alone; living with parents; living with spouse; living with children; and living with other people. How many people living with?], **social support** [10,11], and **social capital** (cognitive and structural dimensions) [12,13].

**Social support**

We assessed social support with the ENRICHD Social Support Instrument (ESSI) [10,11]. A higher total score indicates higher availability of social support.

**Social capital**

To determine social capital, we partly referred to the Integrated Questionnaire for the Measurement of Social Capital (SC-IQ) [12]. It is common practice in social capital research to distinguish between structural and cognitive dimensions [13].

We focused on cognitive social capital using the following SC-IQ items: (1) “Can most people be trusted?”; (2) “Does one have to be alert or is someone likely to take advantage of you?”; and (3) “Are most people willing to help if you need it?” Responses were selected from four categories: “strongly disagree,” “disagree,” “agree,” and “strongly agree.” For the three questions, cognitive social capital was defined as high when there were two or more responses of “agree” or “strongly agree” to question (1), “disagree” or “strongly disagree” to question (2), and “agree” or “strongly agree” to question (3).

We also focused on structural social capital using the following SC-IQ items: “How often do you participate in community organizations, self-help groups, charities, volunteer groups, or religious gatherings?” The response was selected from four categories: “not at all/ very seldom,” “sometimes,” “less than once a week,” and “more than once a week.” Structural social capital was defined as high when the response was “more than once a week.”

1. **Internal validation and external validation results of SZ classifier model**

**Table 1.** Confusion matrix of internal validation for SZ classifier model.

|  |  | **Observed cases** | |
| --- | --- | --- | --- |
|  |  | **Schizophrenia** | **Non-schizophrenia** |
| **Predicted cases** | **Schizophrenia** | 24 | 11 |
|  | **Non-schizophrenia** | 19 | 346 |
|  | **Total** | 43 | 357 |

**Table 2.** Confusion matrix of external validation for SZ classifier model: results of all cases.

|  |  | **Observed cases** | |
| --- | --- | --- | --- |
|  |  | **Schizophrenia** | **MDD, BD, and OCD** |
| **Predicted cases** | **Schizophrenia** | 46 | 50 |
|  | **MDD, BD, and OCD** | 15 | 39 |
|  | **Total** | 61 | 89 |

**Table 3.** Confusion matrix of external validation for SZ classifier model: results of non-schizophrenia cases.

|  |  | **Observed cases** | | |
| --- | --- | --- | --- | --- |
|  |  | **MDD** | **BD** | **OCD** |
| **Predicted cases** | **Schizophrenia** | 31 | 19 | 0 |
|  | **Non-schizophrenia** | 25 | 13 | 1 |
|  | **Total** | 56 | 32 | 1 |

**References**

1. Rakuten Insight, Inc. Rakuten Insight. https://insight.rakuten.co.jp/en/aboutus.html [accessed May 30, 2024].
2. Kusama T, Kiuchi S, Takeuchi K, et al. Information usage and compliance with preventive behaviors for COVID-19: a longitudinal study with data from the JACSIS 2020/JASTIS 2021. Healthcare. 2022;10(3):521. doi: 10.3390/healthcare10030521
3. Wakabayashi M, Sugiyama Y, Takada M, Kinjo A, Iso H, Tabuchi T. Loneliness and increased hazardous alcohol use: data from a nationwide internet survey with 1-year follow-up. Int J Environ Res Public Health. 2022;19(19):12086. doi: 10.3390/ijerph191912086
4. Sasaki R, Ota A, Yatsuya H, Tabuchi T. Gender difference in fear and anxiety about and perceived susceptibility to COVID-19 in the third wave of pandemic among the Japanese general population: a nationwide web-based cross-sectional survey. Int J Environ Res Public Health. 2022;19(23):16239. doi: 10.3390/ijerph192316239
5. The Ministry of Health, Labour and Welfare. The standards for the degree of independent living for elderly and disabled people. 1991. https://www.mhlw.go.jp/file/06-Seisakujouhou-12300000-Roukenkyoku/0000077382.pdf. [accessed Oct 17, 2023] (in Japanese).
6. DeSalvo KB, Bloser N, Reynolds K, He J, Muntner P. Mortality prediction with a single general self-rated health question. J Gen Intern Med. 2006;21(3):267-275. doi: 10.1111/j.1525-1497.2005.00291.x
7. Radloff LS. The CES-D scale. Applied psychological measurement. 1977;1(3):385-401. doi: 10.1177/014662167700100306
8. Kohout FJ, Berkman LF, Evans DA, Cornoni‐Huntley J. Two shorter forms of the CES-D depression symptoms index. J Aging Health. 1993;5(2):179-193. doi: 10.1177/089826439300500202
9. Warttig SL, Forshaw M, South J, White A. New, normative, English-sample data for the short form Perceived Stress Scale (PSS-4). J Health Psychol. 2013;18(12):1617-1628. doi: 10.1177/1359105313508346
10. ENRICHD Investigators. Enhancing recovery in coronary heart disease (ENRICHD) study intervention: rationale and design. Psychosom Med. 2001;63: 747–755. PMID: 11573023.
11. Enhancing Recovery in Coronary Heart Disease Patients (ENRICHD): study design and methods. American Heart Journal. 2000;139(1):1-9. doi: 10.1016/s0002-8703(00)90301-6
12. Grootaert G, Narayan D, Jones VN, Woolcock M. Measuring social capital: an integrated questionnaire. World Bank Publications. January 2004. https://ideas.repec.org/b/wbk/wbpubs/15033.html. [accessed May 30, 2024].
13. Murayama H, Fujiwara Y, Kawachi I. Social capital and health: a review of prospective multilevel studies. J Epidemio. 2012;22(3):179-187. doi: 10.2188/jea.je20110128
